# Treatment of Spinal Cord Injury with Intravenous Immunoglobulin G: Preliminary Evidence and Future Perspectives

**DOI:** 10.1007/s10875-014-0021-8

**Published:** 2014-04-11

**Authors:** Apostolia Tzekou, Michael G. Fehlings

**Affiliations:** 1 Toronto Western Research Institute and Krembil Neuroscience Centre, University Health Network, University of Toronto, Toronto, Canada; 2 Division of Neurosurgery, Toronto Western Hospital, University Health Network, University of Toronto, 399 Bathurst St. Suite 4WW-449, Toronto, ON M5T2S8 Canada

**Keywords:** Spinal cord injury, IVIG, immunomodulation, review, neuroinflammation

## Abstract

Neuroinflammation plays an important role in the secondary pathophysiological mechanisms of spinal cord injury (SCI) and can exacerbate the primary trauma and thus worsen recovery. Although some aspects of the immune response are beneficial, it is thought that leukocyte recruitment and activation in the acute phase of injury results in the production of cytotoxic substances that are harmful to the nervous tissue. Therefore, suppression of excessive inflammation in the spinal cord could serve as a therapeutic strategy to attenuate tissue damage. The immunosuppressant methylprednisolone has been used in the setting of SCI, but there are complications which have attenuated the initial enthusiasm. Hence, there is interest in other immunomodulatory approaches, such as intravenous Immunoglobulin G (IVIg). Importantly, IVIg is used clinically for the treatment of several auto-immune neuropathies, such as Guillain-Barre syndrome, chronic inflammatory demyelinating polyneuropathy (CIPD) and Kawasaki disease, with a good safety profile. Thus, it is a promising treatment candidate for SCI. Indeed, IVIg has been shown by our team to attenuate the immune response and result in improved neurobehavioral recovery following cervical SCI in rats through a mechanism that involves the attenuation of neutrophil recruitment and reduction in the levels of cytokines and cytotoxic enzymes Nguyen et al. (J Neuroinflammation 9:224, 2012). Here we review published data in the context of relevant mechanisms of action that have been proposed for IVIg in other conditions. We hope that this discussion will trigger future research to provide supporting evidence for the efficiency and detailed mechanisms of action of this promising drug in the treatment of SCI, and to facilitate its clinical translation.

SCI is a devastating condition on a physical, psychological, and financial level, with the life-time cost for a 25-year old ranging from $0.7 to $3 million [2]. Currently there are limited pharmacological treatment options to complement surgical intervention in the effort to facilitate functional recovery after SCI, and their efficacy is questionable [3]. Therefore, it is of vital importance to search for new treatment options for this debilitating condition.

It has been established that on a biological level, SCI consists of two processes: the initial mechanical trauma and the secondary pathophysiological events that extend the tissue damage in the penumbra region. The initial trauma is caused by fracture or dislocation of vertebrae, which imposes shear, stretch, laceration and, more commonly contusion and compression on the spinal cord. Soon after the initial hemorrhage and necrosis in the gray matter, the secondary injury takes place. This involves inflammation, additional petechial hemorrhages extending into the white matter, edema and release of coagulation factors and vasoactive amines [4]. These events cause thrombosis, vasospasm and hypoxia in the injured spinal cord, while, at the cellular level, they cause lipid peroxidation, ionic imbalance, free radical formation and glutamatergic excitotoxicity followed by cell death, demyelination and axonal degeneration [5].

The immune response is believed to orchestrate the secondary injury events [6]. The first cell type to be activated immediately following SCI is microglia. They secrete pro-inflammatory cytokines (Tumor Necrosis Factor α (TNFα), interleukin-1 (IL-1) and IL-6) which results in chemokine production and the recruitment of peripheral leucocytes at the injury site. Leukocytes in turn secrete more TNFα and IL-1, which leads to the upregulation of more inflammatory mediators, such as Reactive Oxygen Species (ROS), cytokines, inducible nitric oxide synthase (iNOS), prostaglandin synthase-2, arachidonic acid, proteases and endothelial cell adhesion molecules [7]. By 24 h post-injury, neutrophils reach the lesion site [8, 9]. In addition to cytokines, they also produce matrix metalloproteinase-9 (MMP-9), [10], that act together to loosen the extracellular matrix to enhance leukocyte chemotaxis and extravasation, activate glia and exacerbate neuronal damage [11]. Furthermore, as a result of neutrophil recruitment, there is an increase in the activities of superoxide dismutase and myeloperoxidase (MPO) at the site of injury, which mediate respiratory burst. The neutrophil recruitment declines by 48 h [8, 9], and, as their count is reduced, monocytes start to accumulate at the site of injury. There, they differentiate into macrophages by 72 h and are activated to secrete glutamate, TNFα, IL-1 and IL-6 and activate iNOS [12, 13]. Furthermore, their activation results in the activation of cyclooxynases, leading to production of prostanoids, which have the potential to enhance the secondary injury [14]. The presence of macrophages at the injury epicenter starts to decrease at 7 days post injury. Activated microglia, on the other hand, are found at relatively stable numbers soon after the injury until several weeks later [15].

Although excessive recruitment and activation causes tissue damage, it is important to note that neutrophils and microglia/macrophages are at the same time necessary to promote recovery after SCI [15]. For example, they phagocytose and remove debris. In the case of microglia, it has been suggested that removal of myelin debris (which inhibits axonal regrowth) enhances axonal sprouting [16]. In addition, microglia/macrophages also secrete anti-inflammatory cytokines and neurotrophic factors [15]. Different populations of microglia/macrophages have different phenotypes (polarization states), which can be pro-inflammatory / cytotoxic (M1) or anti-inflammatory / protective (M2), [17] and the balance of these functions may be critical for conferring neuroprotection or further injury of the nervous tissue.

The role of lymphocytes in the pathogenesis of SCI is perhaps even less clear. Following the disruption of Blood-Spinal Cord Barrier (BSCB), B and T cells are exposed to CNS antigens. The autoimmunity resulting from that exposure is believed by some people to be pathological [18]. In contrast, Schwartz and colleagues assert that autoimmunity, such as that conferred by myelin-reactive T cells, is protective [19]. There exists evidence to support both sides of this argument. Again, the beneficial or detrimental activity of the T cells depends on their subtype (cytotoxic – CD8+ or helper – CD4+), the specific time, location and extent of activation. Our understanding of the functions of lymphocytes in the injured nervous system is still incomplete and needs further investigation [15]. For more details on the roles of leukocytes in injury and repair after SCI, the reader is directed to reviews [20, 21].

Yet another example of an aspect of the immune system that may have both beneficial and detrimental effects on recovery after SCI is the Complement system. Complement gets activated after injury and the active fragments get deposited on neurons and oligodendrocytes. Complement inhibition or genetic depletion of factor B, C1q or C3 has been shown to result in improved neurobehavioral outcomes, whilst antagonism of the receptor for C5a results in worse locomotor recovery [22].

This dual role of the immune system in SCI was not always recognized and has become more evident in recent years. Initially it was thought that suppressing the immune response would have a beneficial effect on the outcome after SCI. For example, the immunosuppressant glucocorticoid steroid methylprednisolone used to be the standard of care for acute SCI. However, both its effectiveness in improving motor recovery and its safety are nowadays debated. In particular, it has been concluded that early administration (within the first 8 h after injury) is essential to improve motor recovery, while if administered after 8 h, recovery may be worse. Furthermore, methylprednisolone use has been associated with serious side effects, such as wound infections and pneumonia. [3, 23]. Its mechanism of action is based mainly on the inhibition of post-traumatic lipid peroxidation In addition, functions mediated through glucocorticoid receptor binding suppress the immune system. Since, as recently described, the immune system of SCI patients is already suppressed, [24] the end result of methylprednisolone administration is susceptibility to infections. Therefore, there is a great need to investigate new immunomodulatory approaches which selectively minimize the harmful and augment the beneficial aspects of inflammation, as opposed to immunosuppressive ones which shut it down globally.

Our publication in 2012 demonstrated the effectiveness of early treatment with human IVIg, in improving the recovery of rats after experimental SCI [1]. Importantly, IVIg is believed to modulate the immune response and is used clinically in a variety of conditions with an overall good safety profile. It is composed of human IgG purified from the pooled serum of thousands of donors and its initial use was for the treatment of patients with primary antibody deficiencies. In 1981, the first immunomodulatory property of IgG was discovered (increase of platelet counts in patients with idiopathic thrombocytopenic purpura (ITP)) followed by the establishment of IgG as a treatment for that condition. Subsequently, IgG became an indicated treatment for Kawasaki disease, myasthenia gravis, dermatomyositis, as well as a variety of autoimmune neuropathies, notably Guillain-Barré syndrome, chronic inflammatory demyelinating polyradiculoneuropathy, multi-focal motor neuropathy and stiff person syndrome [25–30]. Furthermore, IgG is currently being investigated as a potential treatment for conditions with similar pathobiology to SCI, such as stroke [31, 32] and multiple sclerosis [33].

Since IVIg seems to be an efficient modulator of neuroinflammation, it was reasonable to examine its effects in SCI. A first report by Gok et al. [34] showed that IgG reduces the levels of myeloperoxidase (MPO) in the injured spinal cord, using a weight drop, thoracic model of injury. MPO is an enzyme found in the azurophilic granules of neutrophils and correlates with the number of neutrophils in the tissue. In addition, IgG was found to correlate with preservation of tissue ultrastructure and locomotor function. However, the route of administration of IgG used in that study was intraperitoneal, which is not highly clinically-relevant. In addition, the locomotor function and tissue ultrastructure was evaluated at only a single time point after injury (24 h) and the long-term effects of IgG were not examined.

In 2012, our team (see paper by Nguyen et al.) [1] described the beneficial effects of IVIg more convincingly in a well-characterized, clinically-relevant model of cervical SCI in the rat, using a modified aneurysm clip [35, 36] to induce a compression/contusion injury. A single dose of 0.4 g/kg IVIg was delivered via the tail vein at 15 min post-injury. The first important finding was that IgG was able to reach the injury site, taking advantage of the compromised BSCB, while it did not cross the BSCB in uninjured animals. There, it was found to co-localize with astrocytes and surround microglia/macrophages. Importantly, IVIg was associated with reduced neutrophil infiltration of the injury site at 24 h post injury, as determined by MPO activity and immunohistochemistry. In addition, it reduced the SCI-mediated upregulation of the expression of matrix metalloproteinase (MMP)-9, an enzyme that degrades the collagen matrix to allow neutrophils to extravasate towards the injury site. IVIg significantly ameliorated the SCI-mediated increase in the levels of pro-inflammatory cytokines IL-1β and IL-6, and the chemokine MCP-1 in the injured spinal cord at 4 h after the trauma. All these anti-inflammatory effects were associated with a reduction in the area of scar and cavity and increased preservation of neural tissue. More importantly, IgG treatment resulted in improved locomotor recovery and coordination in comparison to the vehicle, as evaluated by weekly scoring of the hind-limb function of rats allowed to walk in an open field (BBB scale [37]), and the maximum angle at which they are able to maintain a horizontal position on an inclined plane apparatus [38]. The functional recovery was supported by the enhanced electrophysiological evidence of axonal conduction [1].

Our study convincingly demonstrated the therapeutic potential of IVIg in yet another condition, SCI, for which there is great need of treatment options. The fact that a single, low dose of IgG improved recovery after SCI is impressive. Considering that doses of 0.2–0.4 g/mg body weight are used in the clinic in case of antibody deficiencies, while a high dose of 2 g/kg body weight is used as an immunomodulatory agent in inflammatory disorders, [30, 39] it is exciting to think that there is potentially room for improvement. This can be achieved either by increasing the dose or administering multiple doses. Future preclinical research to establish the dose response as well as the efficiency of administration of IgG at delayed, more clinically-relevant time points is imperative. Furthermore, the mechanism of action of IgG in SCI should be examined before clinical translation is possible.

Despite the increasing list of diseases for which IgG is being used or evaluated, the mechanism(s) of action are poorly understood. IgG is a mixture of antibodies with specific reactivities to infectious agents and autologous antigens, anti-idiotypic antibodies (which neutralize autoantibodies), and other proteins and small molecules. Each of these may be contributing a different effect to the overall mechanism of action of IgG. There are many reviews that summarize the immunomodulatory properties of IVIg [40–42], several of which are relevant to neurological disorders [43] and therefore possibly relevant to SCI. Coming from the pooled plasma of thousands of donors, the different antibody specificities in IVIg can bind and neutralize / block a wide range of cell-surface and soluble targets, therefore mediating a variety of effects. These properties of IgG include neutralization and enhanced clearance of auto-antibodies, interference with the Complement system, modulation of B- and T-cell function, inhibition of leukocyte migration, induction of leukocyte apoptosis and possibly suppression of cytokine levels. In addition to the effects that are mediated through the variable region of individual antibody species, IVIg has been shown to bind to the Fc receptors of phagocytes through the Fc fragment and interfere with phagocytosis or induce the expression of the inhibitory receptor FcγRIIB [25, 44, 45], mechanisms that are of relevance in the case of ITP [46] and Guillain-Barré syndrome [47]. Here we discuss the mechanisms of action of IgG that are potentially relevant to SCI and therefore worth investigating in relation to this condition in the future.

As mentioned, IgG was shown by Nguyen et al. to attenuate neutrophil infiltration in the injured spinal cord. This is important since, as mentioned, neutrophils contribute significantly to pathology. In fact, studies have indicated that neutrophil depletion results in improved functional recovery [48]. It is worth exploring whether the attenuation in the number of neutrophils was caused by a reduced recruitment to the injury site or because of a reduction in the systemic count of neutrophils or if it is a combination of both effects. IgG has been shown to induce apoptosis to neutrophils through binding to sialic acid-binding immunoglobulin-like lectin-9 [49]. The attenuation of SCI-mediated increase in the levels of IL-1β, IL-6, MCP-1, and MMP-9 in the spinal cord shown in our study points towards reduced neutrophil recruitment to the spinal cord without excluding any effect on the total cell count. The effect on cytokines is in agreement with literature showing that IgG modulates the expression of cytokines and their receptors [50, 51]. However the list of cytokines and chemokines presented by Nguyen et al. is small, and in the future it would be interesting to examine how IVIg treatment affects the full cytokine profile (including pro-inflammatory and anti-inflammatory cytokines) following SCI.

One aspect of the recruitment of neutrophils or other leukocytes is the extravasation step. IgG was shown to inhibit rolling and adhesion of leukocytes on the endothelium and therefore their extravasation to the injury site, as shown both in vitro and in vivo by intravital microscopy in two different studies [52, 53]. LaPointe, for instance, showed that IVIg treatment results in reduced leukocyte adhesion by blocking the α4-integrin interaction with vascular cell adhesion molecule-1 (VCAM-1) [53]. The blockage of α4-integrin – VCAM-1 interaction - is the mechanism of function of the therapeutic antibody natalizumab, which is used to treat relapsing-remitting Multiple Sclerosis, a disease for which IVIg is also a treatment option. In addition, α4β1-integrin blockade has been shown to decrease the systemic inflammatory response after SCI [54]. IgG may also inhibit cell adhesion through antibodies to the Arg-Gly-Asp (RGD) motif, which is present on cell surface and matrix proteins and is responsible for the interaction with integrins [55].

The aforementioned intravital microscopy studies did not identify the affected cell types. In addition to neutrophils IVIg may affect the extravasation of other cell types that are important in the pathobiology of SCI. Also, IgG preparations have been demonstrated to contain agonist anti-Fas antibodies, which induce monocyte and lymphocyte apoptosis via a caspase-dependent pathway [56]. Therefore, although the effect of IVIg on macrophage recruitment and activation in the injured spinal cord has not been examined yet, it is possible that it will also be attenuated.

It is plausible that by reducing the recruitment of phagocytes to the injury site and by blocking phagocytosis through the saturation or modulation of the Fcγ receptors, IVIg reduces antigen presentation and therefore the adaptive immune response. The large repertoire of specificities in IVI may also neutralize the newly released CNS antigens following SCI. Similarly, it may neutralize auto-antibodies and auto reactive B-cell receptors, the blockade of which may lead to the suppression of autoimmunity [25]. In addition, it has been suggested that IVIg interacts with auto-reactive T helper cells and down-regulates excessive Th1 or Th2 responses [43]. As mentioned, the role of autoimmunity in SCI is controversial [57, 58], and it is worth investigating how IVIg affects it. In fact, IVIg could serve as a tool to further examine the role of the autoimmune response in recovery after SCI. The above proposed mechanisms are depicted in Fig. 1.

**Fig. 1:**
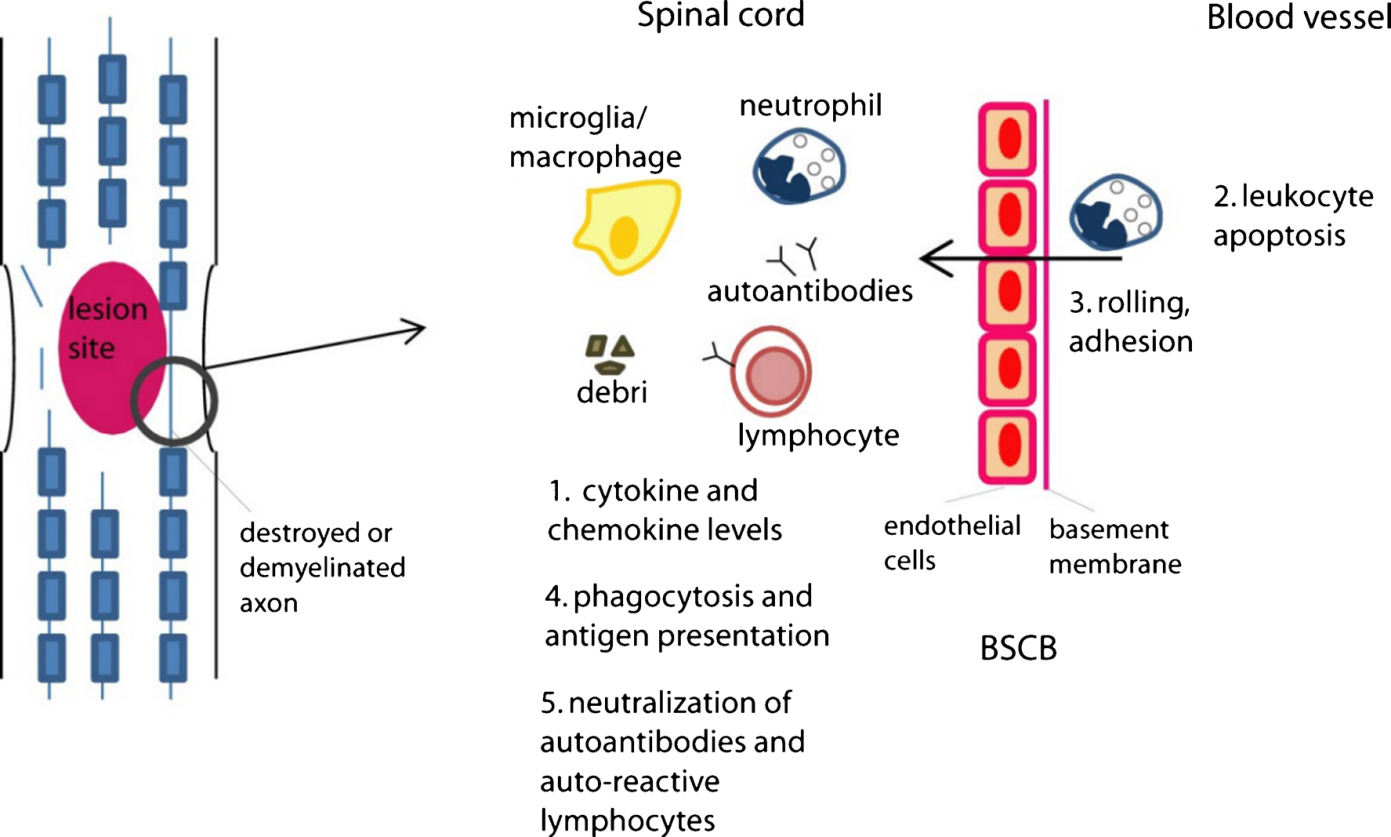
Schematic diagram depicting some of the potential mechanisms of action of IVIg in the treatment of SCI as discussed in the text. Following the neuronal necrosis and axonal destruction and demyelination, the immune system gets activated and leukocytes get recruited in the spinal cord, starting with neutrophils. IVIg may interfere with several steps of the cellular immune response (numbered 1–5). It has been shown to reduce cytokine and chemokine levels, as well as neutrophil recruitment, which may be due to increased leukocyte apoptosis or decreased rolling and adhesion and therefore extravasation. In addition, IVIg has been suggested to interfere with phagocytosis and antigen presentation. Finally, it may interfere with the adaptive immune response by neutralizing CNS antigens, auto-antibodies and auto-reactive lymphocytes

IVIg displays several other immunomodulatory functions that may be relevant to the treatment of SCI. For example, it interferes with the Complement system [22, 59]. IVIg can scavenge the activated complement fragments, such as the anaphylatoxins C3a and C5a and the opsonins C3b and C4b, preventing their signaling / deposition, respectively on host cell targets [43]. As mentioned, IVIg colocalized with astrocytes in the injured spinal cord, but not with microglia. The functional implications of this cellular specificity are currently unknown. This binding may have contributed to the reduced levels of cytokines that were reported. It would be interesting to examine if it affects scar formation. Other systemic or local cellular targets of IVIg should also be explored.

In addition to modulating the immune response, IVIg may exert protective effects on the nervous tissue through additional mechanisms (Table I). For example, IVIg may ameliorate vascular permeability, as indicated by the reduced frequency of attacks in systemic leak capillary syndrome patients [60] and suppression of the increased intestinal vascular permeability and mucosal damage in the gut of mice induced by Clostridium difficile toxin [61]. This is in agreement with the fact that IVIg treatment following reduced the level of MMP-9 in the spinal cord. Therefore, it would be interesting to further investigate its effects on BSCB disruption after SCI in the future, since this is a major contributor of the SCI pathology.

**Table I:** Reported functions of IVIg that may be of relevance to SCI

Immunomodulation of cellular responses (see Fig. 1)
Interference with the Complement system
Amelioration of vascular permeability
Promotion of remyelination
Reduction of neuropathic pain

Demyelination following SCI is another major factor that contributes to pathology. IVIg has been suggested to promote remyelination, although it was later proposed that this effect is mediated by the IgM species of the IVIg preparation which bind to oligodendrocytes [62, 63]. IVIg has also been associated with reduced neuropathic pain (an important problem for SCI patients) in certain conditions [64, 65] (e.g. diabetes [66, 67], via the suppression of the immune-mediated demyelination in the peripheral nervous system [47]. This gives a strong incentive to examine the effect of IVIg on remyelination and neuropathic pain in the future.

In conclusion, IVIg has emerged as a potential pharmacological treatment option for SCI that reduces inflammation and improves the neurobehavioral recovery of rats. The key mechanistic finding in our study was that IVIg ameliorates SCI-induced neutrophil recruitment to the injury site. However, the mechanisms through which this is achieved are not yet known. In addition, as discussed, IVIg is known to modulate the activity of all immune cells and other aspects of the immune response. The mechanisms of action of IVIg in other inflammatory conditions including Guillain Barré syndrome, Kawasaki disease and ITP are likely to apply here. Optimization of the treatment paradigm and exploration of these potential mechanisms in the context of SCI will support the clinical translation of this drug for its use in SCI, which may improve the quality of life of SCI sufferers.
